# A novel optimized surgical strategy for secondary hyperparathyroidism: clean parathyroidectomy combined with autotransplantation and its clinical analysis

**DOI:** 10.3389/fendo.2026.1845268

**Published:** 2026-07-06

**Authors:** Bao-Zhong Yao, Sai-Long Sang, Li Lin, Kun Peng, Hong-Cun Chen, Hong-Lin Li, Dai-Wei Shi, Liang Li, Qi-Ru Xiong

**Affiliations:** 1 Department of Thyroid and Breast Surgery, The Second People’s Hospital of Hefei (Anhui Medical University Affiliated Hefei Hospital), Hefei, Anhui, China; 2 Department of General Surgery, The Second Affiliated Hospital of Anhui Medical University, Hefei, Anhui, China

**Keywords:** 1,25-dihydroxyvitamin D, autotransplantation, clean parathyroidectomy, intraoperative PTH, kidney transplantation, secondary hyperparathyroidism, total parathyroidectomy

## Abstract

**Background:**

Recurrent disease due to ectopic parathyroid tissue remains a significant challenge following conventional total parathyroidectomy with autotransplantation (TPTX+AT) for refractory secondary hyperparathyroidism (SHPT). This retrospective cohort study aimed to evaluate the safety and medium-term efficacy of a standardized, extended surgical approach—clean parathyroidectomy with autotransplantation (CPTX+AT)—which systematically resects the central neck compartment to address this issue. We also clarified the exclusion criteria for kidney transplantation candidates and the application of intraoperative parathyroid hormone (ioPTH) measurement to optimize the surgical strategy.

**Methods:**

A total of 98 patients with uncontrolled SHPT who underwent CPTX+AT between April 2021 and June 2023 were enrolled as the Observation Group, and 80 patients who underwent TPTX+AT between January 2020 and March 2021 served as the Control Group. All patients were excluded from kidney transplantation waiting lists or had no definite kidney transplantation plan at surgery. Perioperative outcomes, biochemical parameters (iPTH, calcium, phosphorus, ALP), clinical symptom resolution, thyroid function, complications, recurrence rates (defined as iPTH >500 pg/mL on two consecutive measurements), and patient-centered hard outcomes (mortality, fracture, cardiovascular events) were analyzed. ioPTH was measured in all operations, and surgical strategies were adjusted according to ioPTH decline trends. Kaplan-Meier curves and log-rank tests were used to assess recurrence-free survival, with all patients followed until a uniform data-collection cut-off of 31 December 2024.

**Results:**

Postoperative biochemical parameters improved significantly in the Observation Group, with complete resolution of clinical symptoms by 3 months postoperatively. The overall complication rates were comparable between the groups (12.24% in the Observation Group vs. 12.50% in the Control Group, p > 0.05). Transient hypocalcemia occurred in 6.12% (6/98) of the Observation Group and 5.00% (4/80) of the Control Group; all patients received high-dose 1,25-dihydroxyvitamin D and calcium supplementation postoperatively, and no permanent hypoparathyroidism was observed in either group. During a median follow-up of 25.0 months (range 18–44) for the Observation Group and 47.0 months (range 45–59) for the Control Group, no recurrence was detected in the Observation Group (0/98), while the Control Group had a recurrence rate of 5.0% (4/80) (log-rank *p* = 0.026). Exploratory analysis showed no significant between-group differences in mortality (4.1% vs. 5.0%), fracture (0% vs. 0%), cardiovascular events (3.06% vs. 2.5%).

**Conclusion:**

CPTX+AT is a safe, feasible, and promising medium-term strategy for uncontrolled SHPT in non-kidney transplantation candidates, achieving durable biochemical and symptomatic improvement with no recurrence detected during a median follow-up of 25 months. High-dose 1,25-dihydroxyvitamin D supplementation effectively reduces the risk of severe hypocalcemia, and ioPTH measurement is key to ensuring surgical success. CPTX+AT is particularly suitable for patients at high risk of recurrence due to ectopic parathyroid tissue.

## 1 Introduction

Secondary hyperparathyroidism (SHPT) is a prevalent and serious complication of end-stage chronic kidney disease (CKD) (1), characterized by abnormal parathyroid gland hyperplasia and excessive secretion of parathyroid hormone, leading to disordered calcium-phosphorus metabolism, renal osteodystrophy, and cardiovascular calcification (2). Dialysis and pharmacotherapy (phosphate binders, vitamin D analogs, calcimimetics) are the first-line interventions for end-stage CKD (2), but approximately 4–14% of patients progress to drug-refractory, uncontrolled SHPT (1, 2)—defined as persistent intact parathyroid hormone (iPTH) > 800 pg/mL despite ≥3 months of standardized medical therapy, accompanied by clinical symptoms such as intractable bone pain, pruritus, or pathological fractures (3). For these patients, surgical intervention is a definitive therapeutic modality, as it not only achieves durable remission of metabolic abnormalities but also effectively reduces mortality risk associated with SHPT complications (4–6).

Currently, three mainstream surgical approaches are widely used for drug-refractory SHPT: subtotal parathyroidectomy (SPTX), total parathyroidectomy (TPTX), and total parathyroidectomy with autotransplantation (TPTX+AT). Among them, TPTX+AT is the most commonly adopted strategy in many clinical centers due to its balance of efficacy and safety (7)—it eliminates hyperplastic parathyroid tissue to control SHPT while preserving parathyroid function via autotransplantation, thus reducing the risk of permanent hypoparathyroidism (8). Autologous parathyroid tissue is typically implanted in easily accessible sites such as the forearm brachioradialis muscle, enabling subsequent monitoring and minimally invasive resection of hyperfunctional grafts if recurrence occurs (9).

However, a major limitation of conventional TPTX+AT is the relatively high postoperative recurrence rate, which has been reported to be 2–20% in clinical studies (10–12). Residual ectopic parathyroid tissue is a key contributor to this recurrence. According to an anatomical meta-analysis by Taterra et al., the central neck compartment harbors approximately 30.5% of cervical ectopic glands, accounting for roughly 22% of all residual ectopic parathyroid tissue when including mediastinal locations (13). Furthermore, hyperplasia of the autotransplanted graft itself constitutes another significant source of recurrence. A systematic review by Richards et al. noted that graft-dependent recurrences were found in a wide range of 25–100% of patients who experienced disease relapse after TPTX+AT (14). Ectopic parathyroid glands result from anatomical variations and embryological developmental anomalies, and their elusive localization makes complete resection difficult during conventional surgery (15, 16). Chinese scholars proposed the “seed, environment, soil” hypothesis and introduced the innovative Purge Parathyroidectomy (PPTX) strategy (17), which emphasizes radical resection of parathyroid tissue and surrounding fibrofatty tissue. Early studies by the Vienna group supported systematic central compartment clearance (18), but this concept was further refined in the PPTX strategy (17).

Our clean parathyroidectomy with autotransplantation (CPTX+AT) is an optimized evolution of these radical dissection strategies, with more precise anatomical boundaries for central neck compartment resection and selective superior thymus resection to balance recurrence reduction and surgical safety. Prior to April 2021, our institution used conventional TPTX+AT for SHPT, with a postoperative recurrence rate of approximately 5%. To improve clinical outcomes, we developed and implemented the standardized CPTX+AT technique in April 2021, which involves en bloc resection of all fibrofatty tissue in the bilateral central neck compartment to ensure the removal of all *in situ* and ectopic parathyroid tissue. Preoperative localization relied on neck ultrasound and 99mTc-MIBI SPECT/CT, which are standard in most clinical centers but have lower sensitivity for small or deeply located ectopic glands compared with 18F-fluorocholine PET/CT.

Notably, all patients included in this study were non-kidney transplantation candidates, as TPTX is not recommended for patients with an immediate intention of kidney transplantation ^3.^ Intraoperative parathyroid hormone (ioPTH) measurement was used as the core indicator to evaluate the completeness of parathyroidectomy in all operations (19). This retrospective cohort study aimed to formally evaluate the safety, feasibility, and medium-term efficacy of CPTX+AT for uncontrolled SHPT, and to analyze patient-centered hard outcomes to provide high-quality clinical evidence for its clinical application.

## 2 Materials and methods

### 2.1 Study design and sample size calculation

This was a single-center, retrospective, non-concurrent cohort study (two groups enrolled in different time periods) conducted in accordance with the Strengthening the Reporting of Observational Studies in Epidemiology (STROBE) guidelines. Although the retrospective design precluded a formal non-inferiority hypothesis test, we performed an *a priori* sample size calculation using a non-inferiority framework to ensure adequate power for the primary outcome comparison. The primary outcome was the postoperative recurrence rate of SHPT. Based on our institutional historical data and published literature (10), the expected 2-year recurrence rate of TPTX+AT was set at 10% (*P* control = 0.10). With a non-inferiority margin (Δ) of 10 percentage points, a one-sided alpha of 0.05, and a power of 80%, the required sample size was 76 patients per group. Accounting for a 5% potential loss to follow-up, we aimed to enroll at least 80 patients in each group. The final enrollment (98 in the CPTX+AT group and 80 in the TPTX+AT group) met and exceeded this target. The follow-up protocol was anchored to the date of surgery; all patients were followed until the uniform data-collection closing date of 31 December 2024. As a result, the available follow-up duration was shorter for the later-enrolled Observation Group (range 18–44 months) and longer for the earlier-enrolled Control Group (range 45–59 months).

### 2.2 Study population

Patients with uncontrolled SHPT who underwent CPTX+AT at the Second People’s Hospital of Hefei (Anhui Medical University Affiliated Hefei Hospital) from April 2021 to June 2023 were enrolled as the Observation Group (n = 98). For comparative analysis, 80 patients with uncontrolled SHPT who underwent TPTX+AT in the same department from January 2020 to March 2021 were retrospectively included as the Control Group (n = 80). All patients received regular dialysis (hemodialysis or peritoneal dialysis) and were managed by the same team of senior endocrine surgeons with >10 years of experience to ensure consistency in surgical technique and perioperative management.

#### 2.2.1 Inclusion criteria

Eligible patients met all the following criteria:

① Biochemical/symptomatic criteria: Serum iPTH > 800 pg/mL with hyperphosphatemia (>1.63 mmol/L) and/or hypercalcemia (>2.5 mmol/L) despite optimized medical therapy; or serum iPTH 500–800 pg/mL with severe clinical manifestations (refractory bone/joint pain, pruritus, pathological fracture, progressive ectopic calcification, or hemoglobin < 90 g/L).
② Imaging criteria: Neck ultrasound showing ≥1 parathyroid gland with diameter >1 cm and rich vascularity (or volume ≥500 mm³), or ⁹⁹^m^Tc-MIBI SPECT/CT showing parathyroid hyperdense foci. 18F-fluorocholine PET/CT was not routinely available at our center during the study period.
③ Surgical fitness: No absolute contraindications to general anesthesia or neck surgery.
④ Non-kidney transplantation candidate: No intention or definite plan for kidney transplantation within 6 months of surgery.

#### 2.2.2 Exclusion criteria

Patients were excluded if they had any of the following:

① Uncontrolled hypertension (blood pressure > 180/110 mmHg despite ≥2 weeks of standardized treatment).
② Active systemic infection or inflammation.
③ Coagulopathy (INR > 1.5, aPTT > 1.5× upper limit of normal [ULN], or platelets < 50×10⁹/L without correction).
④ Severe neck pathology precluding safe surgical access.
⑤ Severe comorbidities (NYHA class IV cardiac dysfunction, FEV₁ < 30% predicted pulmonary function, dementia, stroke within 3 months), metastatic malignancy, or other conditions with unacceptable perioperative risk.
⑥ A history of parathyroid surgery for SHPT.
⑦ Being on the kidney transplantation waiting list or having a definite kidney transplantation plan within 6 months.

### 2.3 Preoperative preparation

All patients underwent comprehensive preoperative assessments to exclude anesthesia and surgical contraindications, including:

1. Laboratory tests: Complete blood count, liver and kidney function, coagulation profile, serum electrolytes (calcium, phosphorus, magnesium), and iPTH measurement.
2. Imaging studies and cardiopulmonary evaluation: Preoperative parathyroid localization relied on neck ultrasound and ⁹⁹^m^Tc-MIBI SPECT/CT, which are standard in most clinical centers but have lower sensitivity for small or deeply located ectopic glands compared with ¹⁸F-fluorocholine PET/CT. Because ¹⁸F-fluorocholine PET/CT was not available at our institution during the study period, the preoperative identification of ectopic glands may have been incomplete. Cardiopulmonary function was assessed by electrocardiogram, echocardiography, and pulmonary function tests when clinically indicated.

Dialysis and electrolyte management: All patients received heparin-free hemodialysis (4 hours) or standard peritoneal dialysis on the day before surgery to optimize electrolyte balance and reduce intraoperative bleeding risk. Preoperative oral calcium supplementation was administered as needed to maintain serum calcium at 2.1–2.3 mmol/L.

### 2.4 Surgical procedures

All surgeries were performed under general anesthesia with endotracheal intubation. ioPTH was measured in all operations (baseline before incision and 20 minutes after resection of the final parathyroid gland), and a ≥90% decline in ioPTH was defined as surgical success (19); additional exploration was performed if the decline was insufficient.

#### 2.4.1 Conventional TPTX+AT (control group)

A standard cervical transverse incision was made to expose the thyroid and parathyroid glands. All identified hyperplastic parathyroid glands were completely resected under direct vision. No systematic central neck compartment dissection or fibrofatty tissue resection was performed. Autotransplantation was conducted by implanting minced parathyroid tissue (8 fragments, 1 mm³ each) into the forearm brachioradialis muscle of the non-fistulous side.

#### 2.4.2 CPTX+AT (observation group)

**Figure 1:**
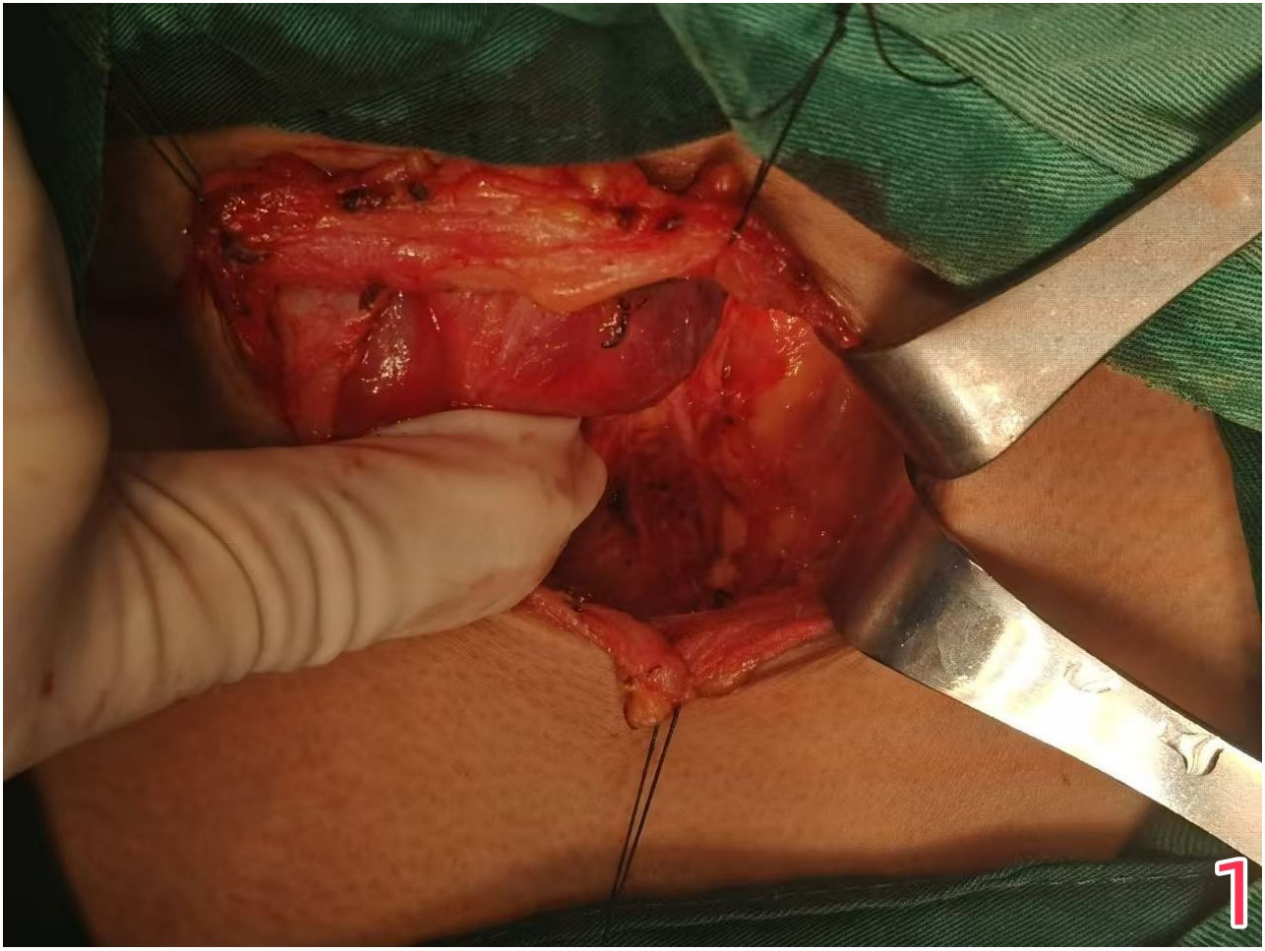
Postoperative left central zone.

**Figure 2:**
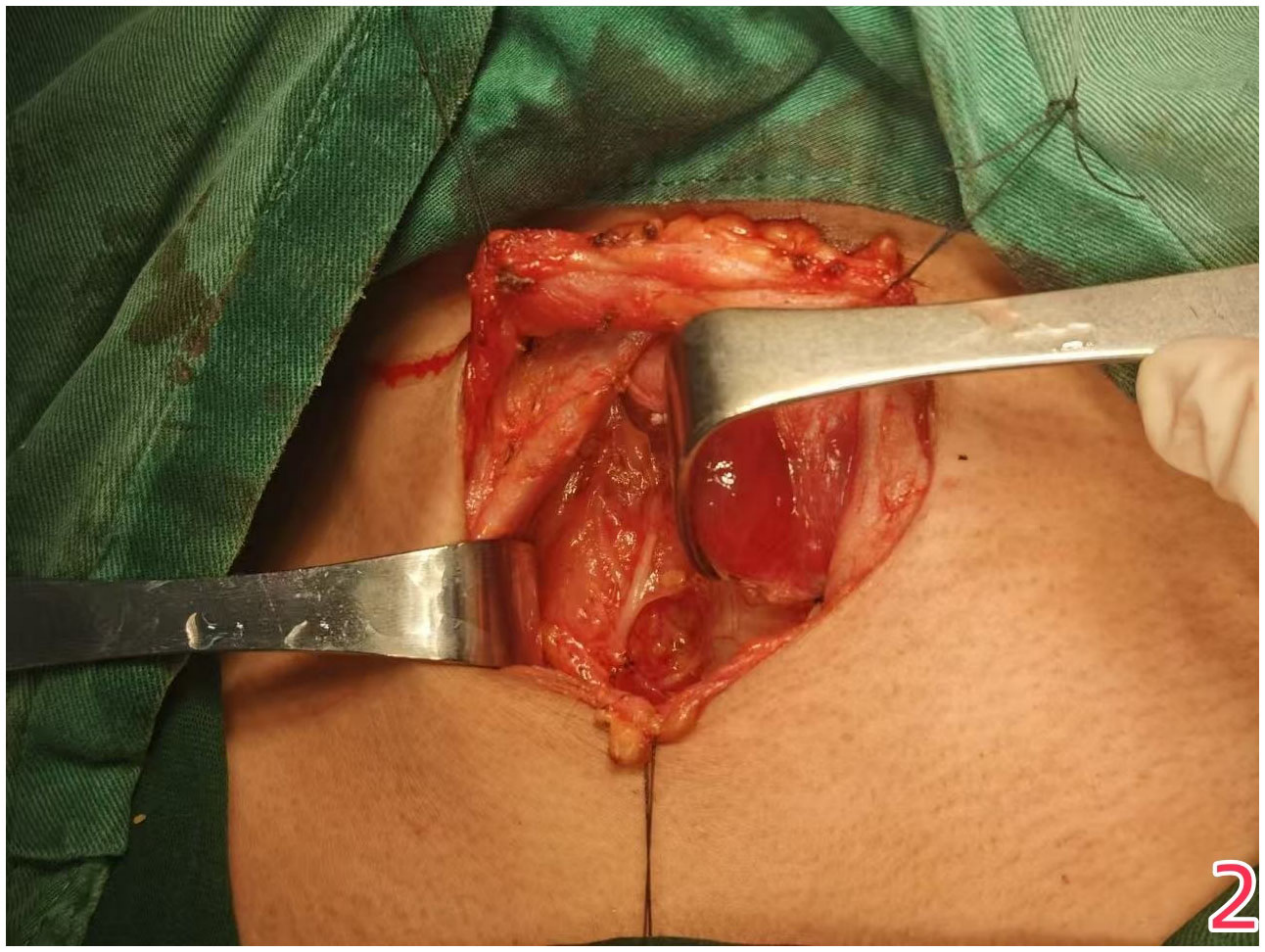
Postoperative right central zone.

**Figure 3:**
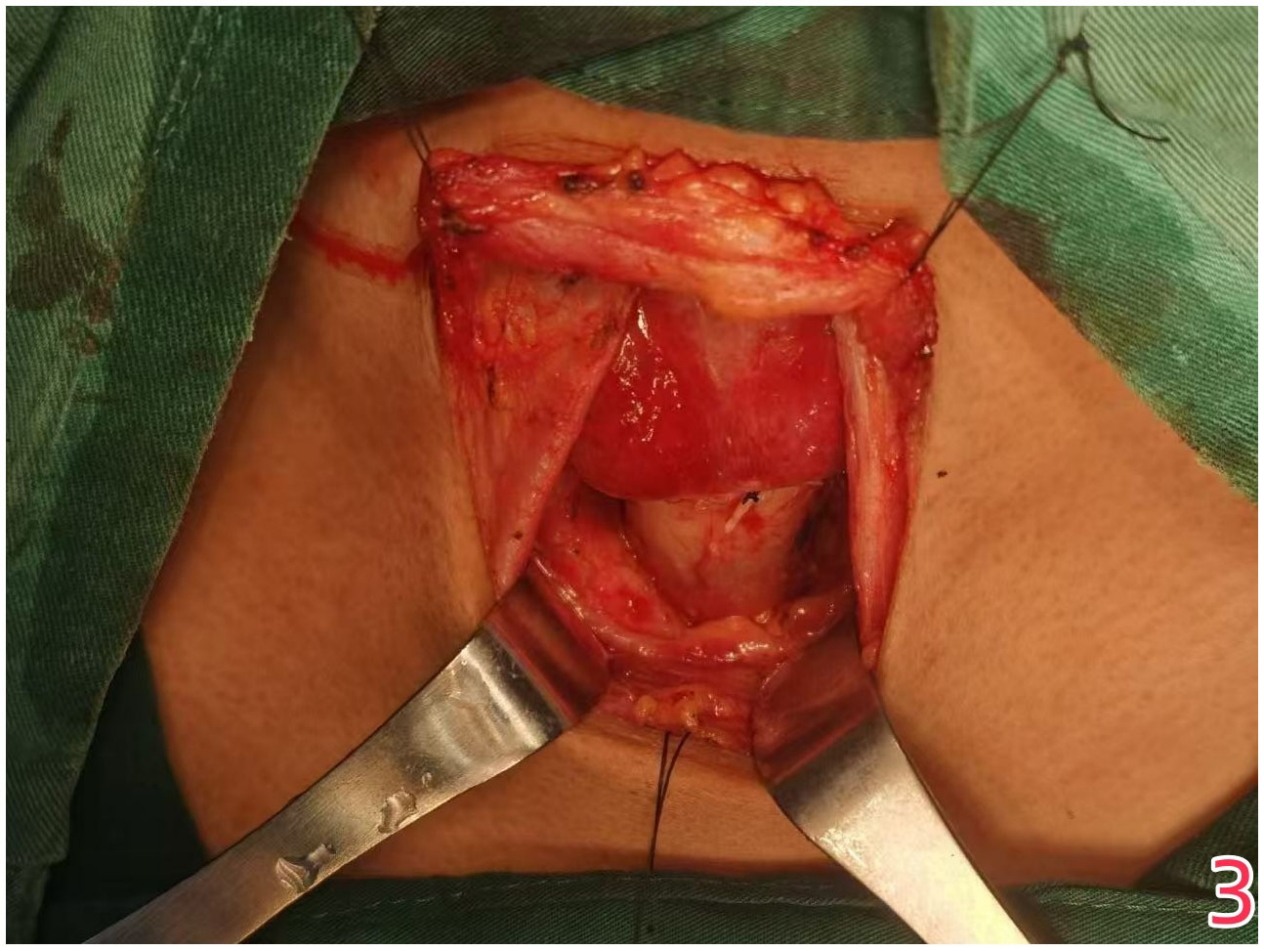
Postoperative pre-tracheal surgery.

**Figure 4:**
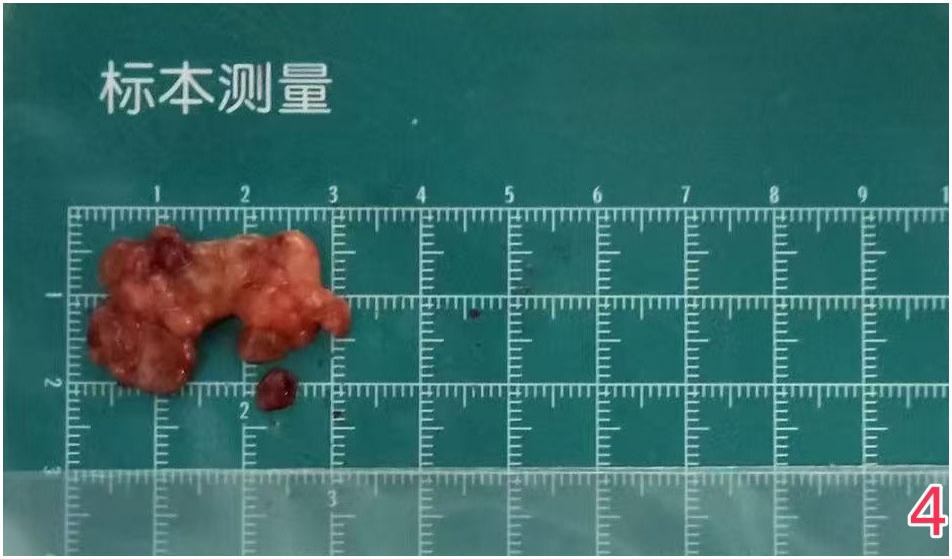
Fibro-fatty tissue in bilateral central zones.

**Figure 5:**
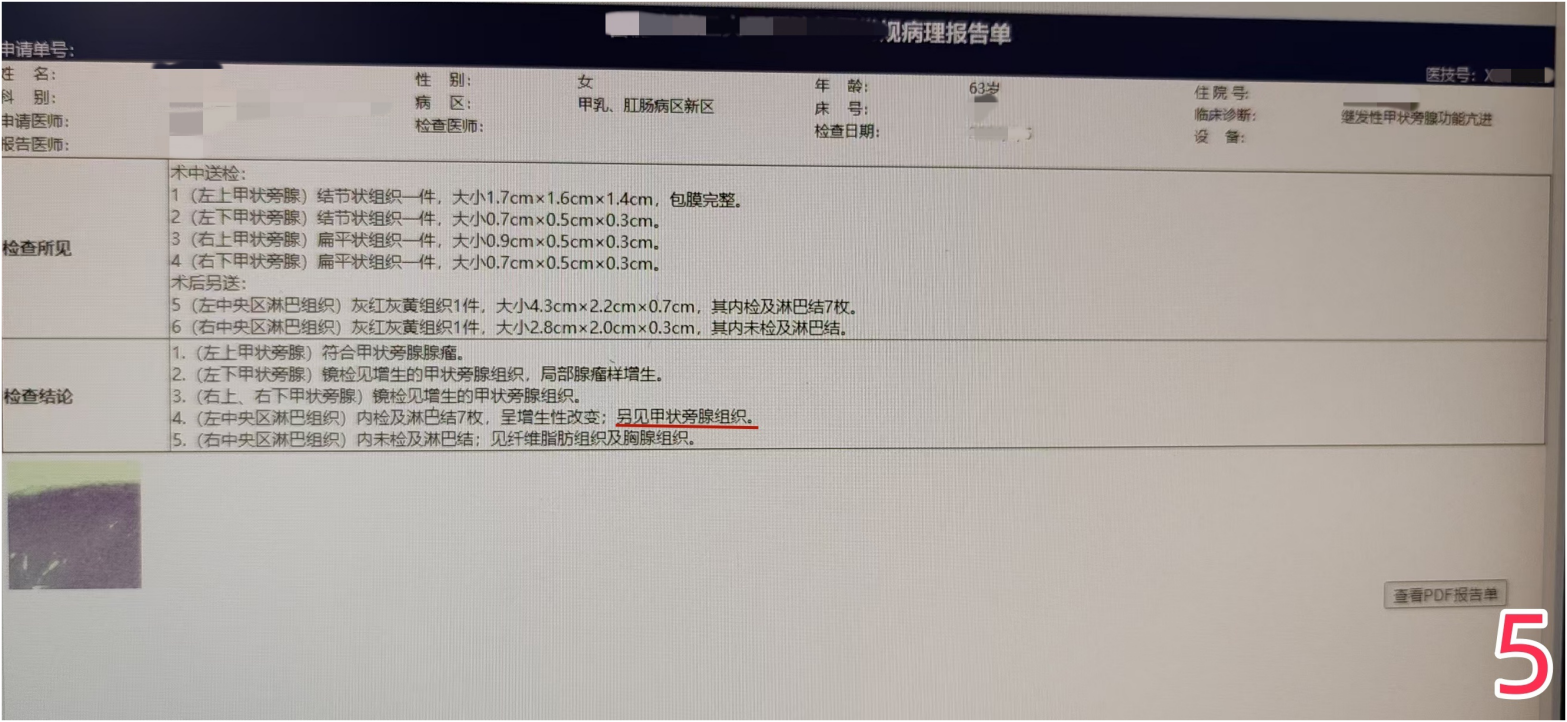
Postoperative pathology results confirming ectopic parathyroid glands.

CPTX+AT included all core steps of conventional TPTX+AT, with the key difference being systematic en bloc resection of the bilateral central neck compartment fibrofatty tissue (including the superior half of the thymus) on the basis of complete *in situ* parathyroid gland resection. The specific steps were as follows:

① Surgical approach and parathyroidectomy: A 4–5 cm low-collar curvilinear incision was made; the platysma and cervical fascia were incised, and the strap muscles were retracted laterally to expose the thyroid gland. The hyperplastic superior and inferior parathyroid glands were identified and resected en bloc with surrounding fat pads, with meticulous preservation of the RLN and thyroid blood supply. Resected parathyroid tissue was immediately immersed in 4 °C Ringer’s solution for autotransplantation.
② ioPTH measurement: Baseline ioPTH (iPTH₀) was measured before skin incision, and a second measurement (iPTH₂₀) was obtained 20 minutes after resection of all visible parathyroid glands. Extended exploration was performed for insufficient ioPTH decline.
③ Central neck compartment dissection: Bilateral RLNs were identified and fully dissected along their entire course with IONM to confirm neural integrity. En bloc resection of all fibrofatty tissue in the bilateral central neck compartment (anatomical boundaries: superiorly the hyoid bone, laterally the carotid sheaths, inferiorly the suprasternal notch, deeply the prevertebral fascia) and the superior half of the thymus was performed. All resected tissue was sent for pathological examination.
④ Autotransplantation: A 2 cm longitudinal incision was made on the forearm brachioradialis muscle of the non-fistulous side; parathyroid tissue confirmed as normal/hyperplastic by frozen section was minced into 8 fragments (1 mm³ each) and implanted into a muscular pocket, which was closed with 4–0 silk sutures.
⑤ Wound closure: A closed-suction drain was placed after meticulous hemostasis, and the wound was closed in layers with a light-pressure dressing applied (**Figures 1**–**5**).

### 2.5 Observation indicators and follow-up

All patients were followed up until 31 December 2024. Follow-up assessments were conducted at 1 week, 1, 3, 6 months postoperatively, and every 3 months thereafter, including clinical symptom evaluation, laboratory tests, and adverse event recording. Blood samples were collected from the non-grafted limb to avoid interference with graft function assessment.

#### 2.5.1 Perioperative and biochemical indicators

Operative time, intraoperative bleeding volume, postoperative drainage volume, and hospitalization time were recorded. Serum levels of iPTH, calcium, phosphorus, ALP, FT3, FT4, and TSH were measured at each follow-up time point. All patients received a standardized postoperative supplementation protocol: high-dose oral 1,25-dihydroxyvitamin D (0.5–1.0 μg/d) and elemental calcium (1000–2000 mg/d) starting on the first postoperative day; intravenous calcium gluconate (1–2 g/d) was added for patients with mild hypocalcemia (serum calcium < 2.0 mmol/L) until serum calcium returned to 2.1–2.3 mmol/L.

#### 2.5.2 Clinical symptom resolution

Preoperative and postoperative clinical symptoms (arthralgia, pruritus, muscle weakness) were evaluated using the VAS (0 = no symptom, 10 = severe symptom). The resolution rate of symptoms was calculated at each follow-up time point.

#### 2.5.3 Complications and recurrence

Perioperative complications were classified according to the Clavien-Dindo Classification System. Recurrence of SHPT was defined as serum iPTH > 500 pg/mL on two consecutive measurements (≥4 weeks apart). Recurrence site (native neck vs forearm autograft) was identified by imaging and Casanova test (differential PTH sampling between grafted and non-grafted arms) in all recurrent patients ¹⁰. The median recurrence time was recorded for recurrent patients.

#### 2.5.4 Patient-centered hard outcomes

Mortality, pathological fracture, cardiovascular events (non-fatal myocardial infarction, stroke), and kidney transplantation rate were recorded during the follow-up period to evaluate the long-term clinical benefits of CPTX+AT. The causes of death were documented and classified as SHPT/surgery-related or unrelated.

### 2.6 Statistical analysis

Statistical analyses were performed using IBM SPSS Statistics 28.0. Continuous variables with a normal distribution were expressed as mean ± standard deviation and compared using the independent samples t-test; non-normally distributed continuous variables were expressed as median (interquartile range, P25–P75) and compared using the Mann-Whitney U test. Categorical variables were expressed as n (%) and compared using the χ² test or Fisher’s exact test (for small sample sizes). Recurrence-free survival was analyzed using the Kaplan-Meier method with the Log-rank test. A two-sided p < 0.05 was considered statistically significant. Multivariable adjustment or propensity score matching was not performed due to the low event rate and retrospective design.

## 3 Results

### 3.1 Perioperative characteristics

All patients in both groups achieved surgical success according to the ioPTH criterion (≥90% decline from baseline), with a mean ioPTH reduction of 91.3% ± 3.5%. In the Control Group, three patients required additional exploration due to insufficient ioPTH decline, and residual parathyroid tissue was identified and resected in all three cases.

The Observation Group had a significantly longer operative time than the Control Group (95 ± 26 min vs. 62 ± 18 min, *p* < 0.05), which was attributed to the additional central neck compartment dissection and ectopic parathyroid gland resection. No significant differences were observed between the two groups in age, gender, body mass index, duration of primary illness, intraoperative bleeding volume, postoperative drainage volume, or mean hospitalization time (all *p* > 0.05). All patients achieved satisfactory cervical wound healing and cosmetic outcomes by one month postoperatively (**Table 1**).

**Table 1:** Perioperative characteristics of the CPTX+AT and TPTX+AT cohorts.

Item	Group		*P value*
	CPTX+AT(n=98)	TPTX+AT(n=80)	
Age (years)	54.08 ± 11.10	49.36 ± 8.25	*>0.05*
Gender(male/female, n)	66/32	44/36	*>0.05*
BMI (kg/m²)	24.59 ± 4.38	26.68 ± 3.64	*>0.05*
Duration of primary illness [M (P25, P75)](years)	11.00(5.00,28.00)	9.00(6.00,18.00)	*>0.05*
Operative time[min]	95 ± 26	62 ± 18	*<0.05*
Intraoperative bleeding volume[mL]	25 ± 5	21 ± 3	*>0.05*
Postoperative hospitalization (days)	5.1 ± 0.7	4.6 ± 1.2	*>0.05*
Postoperative drainage volume (mL)	55 ± 23	42 ± 18	*>0.05*
**Overall complications**	12/98 (12.24%)	10/80 (12.50%)	0.96
**Grade I**	6/98 (6.12%)	4/80 (5.00%)	0.75
Transient hypocalcemia	6/98 (6.12%)	4/80 (5.00%)	
**Grade II**	6/98 (6.12%)	4/80 (5.00%)	0.71
Transient hypoparathyroidism	4/98 (4.08%)	4/80 (5.00%)	
Subcutaneous effusion	2/98 (2.04%)	0/80 (0.00%)	
**Grade III-V**	0/98 (0.00%)	0/80 (0.00%)	1.00
*In situ* parathyroid glands excised [n (%)]			
3	6/98 (6.12%)	4/80 (5.00%)	*>0.05*
4	90/98 (91.84%)	76/80 (95.00%)	*>0.05*
5	2/98 (2.04%)	0/80 (0.00%)	*>0.05*
Hyperplasia confirmed [n/N (%)]			
*In situ* parathyroid glands	388/388 (100.00%)	316/316 (100.00%)	*>0.05*
Ectopic parathyroid glands	10/22 (45.45%)	–	*N/A*
Follow-up duration, median (range), months	25.0 (18–44)	47.0 (45–59)	*<0.05*
SHPT recurrence during a median follow-up period [n (%)]	0/98 (0.00%)	4/80 (5.00%)	*0.026*

BMI, body mass index; SHPT, secondary hyperparathyroidismt; N/A, not applicable.

The bold values indicate statistically significant differences (P < 0.05).

In the Observation Group, a total of 410 parathyroid glands were excised, comprising 388 *in situ* hyperplastic glands and 22 ectopic glands identified within the resected central neck compartment tissue. In the Observation Group, preoperative imaging (ultrasound and 99mTc-MIBI SPECT/CT) identified 16 of 22 ectopic glands subsequently confirmed intraoperatively and pathologically, yielding a sensitivity of 72.7% (16/22). The remaining 6 ectopic glands (27.3%) were not detected on preoperative imaging and were discovered during systematic central neck compartment dissection. All 22 ectopic glands were confirmed by postoperative pathology, with hyperplasia identified in 10 (45.45%). The number of *in situ* parathyroid glands excised was comparable between the two groups (all *p* > 0.05). No patient was lost to follow-up in either group and complete follow-up data were available for all 98 patients in the Observation Group and all 80 patients in the Control Group. The median follow-up time was 25.0 months (range: 18–44 months) for the Observation Group and 47.0 months (range: 45–59 months) for the Control Group. This difference was statistically significant (P<0.05) due to the non-concurrent enrollment, and the shorter follow-up in the CPTX+AT group should be regarded as an important limitation that may reduce the detection of late recurrence. Additionally, because the two groups were enrolled in non-consecutive time periods, the possibility of temporal bias cannot be excluded.

### 3.2 Postoperative biochemical parameters

In the Observation Group, serum iPTH, calcium, phosphorus, and ALP levels changed significantly at all postoperative time points compared with preoperative levels (all *p* < 0.001, **Table 2**). iPTH and calcium reached their nadirs on the first postoperative day, phosphorus normalized by 1 month postoperatively, and ALP declined gradually over 6 months—all biochemical parameters stabilized within the normal range by 6 months postoperatively and remained stable during subsequent follow-up.

**Table 2:** Perioperative changes in laboratory indicators in 98 patients with SHPT who underwent CPTX+AT [mean ± standard deviation or median (interquartile range)].

Time point	iPTH (pg/mL)	Serum calcium (mmol/L)	Serum phosphorus (mmol/L)	ALP (U/L)
Preoperative	1845.78(930.79,2846.23)	2.45 ± 0.213	2.05 ± 0.414	360.60(171.73,652.55)
1 week postoperative	18.05(2.30, 48.58)	1.98 ± 0.25	1.22 ± 0.32	139.40(98.20, 271.95)
1 month postoperative	47.95(18.28,187.18)	2.09 ± 0.292	1.18 ± 0.383	184.80(96.65,309.75)
3 months postoperative	58.90(22.20,253.95)	2.21 ± 0.288	1.41 ± 0.485	190.25(89.70,288.60)
6 months postoperative	63.05(38.03,299.40)	2.17 ± 0.192	1.67 ± 0.491	164.90(90.03,322.98)
F-value	75.56	24.595	35.606	73.333
*P* value	<0.001	<0.001	<0.001	<0.001

iPTH, intact parathyroid hormone; ALP, alkaline phosphatase; postop, postoperative.

### 3.3 Clinical symptom resolution

All 98 patients in the Observation Group experienced significant alleviation of clinical symptoms at all follow-up time points (all *p* < 0.05, **Table 3**). VAS scores for arthralgia decreased significantly by 1 week postoperatively (*p* < 0.001), and all symptoms (arthralgia, pruritus, muscle weakness) resolved completely by 3 months postoperatively, with no recurrence of symptoms during the follow-up period.

**Table 3:** Dynamic changes in clinical symptom status in 98 patients with SHPT who underwent CPTX+AT [n (%)].

Follow-up time point	Arthralgia[n (%)]	Pruritus[n (%)]	Muscle weakness[n (%)]
Preoperative	98(100.0)	64(65.3)	56(57.1)
1 week postoperative	24(24.5)	42(42.9)	34(34.7)
1 month postoperative	12(12.2)	10(10.2)	14(14.3)
3 months postoperative	0(0.0)	0(0.0)	0(0.0)
6 months postoperative	0(0.0)	0(0.0)	0(0.0)
χ² value	173.33	70.5	55.9
P value	<0.001	<0.001	<0.001

### 3.4 Postoperative thyroid function

In the Observation Group, 96 patients (97.96%) had normal FT3, FT4, and TSH levels preoperatively and at all postoperative follow-up time points (1 week, 1, 3, 6 months). Two patients (2.04%) had mild preoperative FT4 elevation and TSH reduction, which resolved spontaneously by 3 months postoperatively. No significant perioperative changes in thyroid function parameters were observed (all *p* > 0.05, **Table 4**), indicating that CPTX+AT did not affect thyroid function.

**Table 4:** Longitudinal changes in thyroid function parameters (FT₃, FT₄, TSH) before and after CPTX+AT in 98 patients with SHPT (x ± s).

Time point	FT3 (pg/mL)	FT4 (pg/mL)	TSH (μIU/mL)
Preoperative	2.55 ± 0.43	0.84 ± 0.18	2.14 ± 1.23
1 month postoperative	2.59 ± 0.54	0.85 ± 0.14	2.70 ± 1.22
3 months postoperative	2.48 ± 0.28	0.82 ± 0.20	2.34 ± 1.35
6 months postoperative	2.61 ± 0.36	0.88 ± 0.15	2.28 ± 1.18
F-value	0.943	1.926	1.794
*P- value*	0.421	0.127	0.149

### 3.5 Complications and recurrence

The overall complication rates were similar between the two groups (12.24% [12/98] in the Observation Group vs. 12.50% [10/80] in the Control Group, *p* = 0.96), with the vast majority of complications being Clavien-Dindo Grade I or II (no Grade III–V complications occurred in either group, **Table 1**). Transient hypocalcemia was observed in 6.12% (6/98) of the Observation Group and 5.00% (4/80) of the Control Group; transient hypoparathyroidism occurred in 4.08% (4/98) and 5.00% (4/80), respectively (all *p* > 0.05). No permanent hypoparathyroidism, permanent RLN injury, hematoma, or surgical site infection was observed in either group during the follow-up period. However, because routine postoperative laryngoscopy was not performed, the 0% RLN injury rate reflects only clinically evident vocal cord palsy; transient hoarseness or subclinical vocal cord dysfunction cannot be excluded.

No recurrence was detected in the Observation Group during the median 25-month follow-up (0/98), whereas the Control Group had a recurrence rate of 5.0% (4/80), with a statistically significant difference between the groups (*p* = 0.026, **Table 5**). Among the four recurrent cases in the Control Group, the median time to recurrence was 18.5 months (range: 12–26 months). Recurrence site (native neck vs. forearm autograft) was identified by imaging and Casanova test (differential PTH sampling between grafted and non-grafted arms) in all four recurrent patients. Three patients (75%) had recurrence originating from the neck (residual ectopic tissue), and one patient (25%) had graft-dependent recurrence.

**Table 5:** Recurrence of SHPT after different surgical approaches.

Group	Number of cases	Recurrence of SHPT, n (%)	*P* value
CPTX+AT	98	0(0.0)	0.026
TPTX+AT	80	4(5.0)	

Recurrence was defined biochemically (iPTH >500 pg/mL on two consecutive measurements). Median follow-up: 25.0 months (range: 18–44 months) for CPTX+AT vs. 47.0 months (range: 45–59 months) for TPTX+AT.

**Figure 6:**
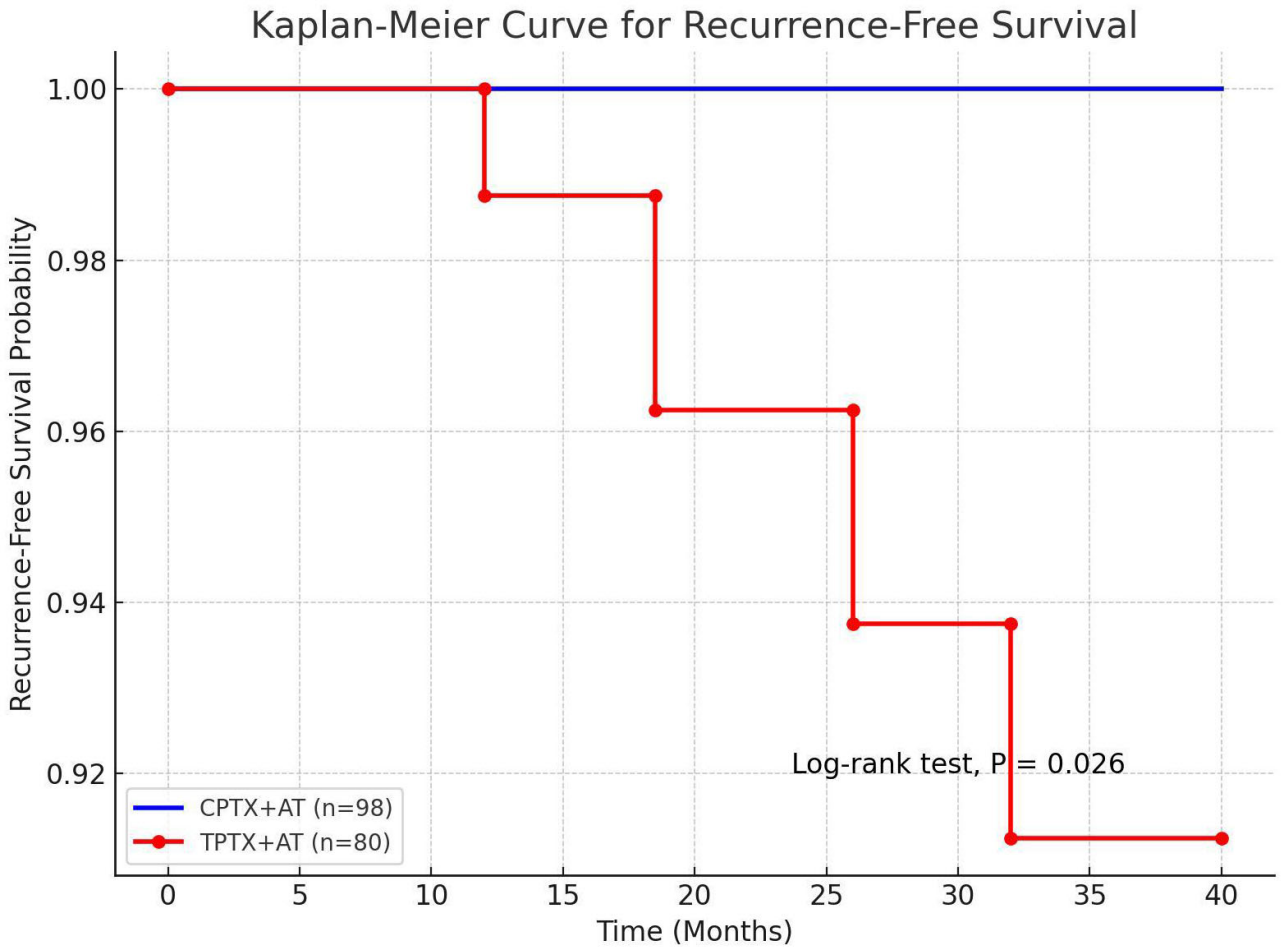
Kaplan-Meier curves comparing recurrence-free survival rates between the two groups.

### 3.6 Recurrence-free survival analysis

Kaplan-Meier analysis showed that the Observation Group had significantly higher recurrence-free survival than the Control Group throughout the follow-up period (Log-rank *p* = 0.026, **Figure 6**). The Observation Group maintained 100% recurrence-free survival over a median follow-up of 25.0 months, while the Control Group had a gradual decline, with 4 recurrence events recorded at a median of 18.5 months postoperatively. Cox regression analysis was not performed due to the small number of events (n = 4) and the absence of events in the CPTX+AT group, resulting in insufficient statistical power. Of note, the median follow-up of 25.0 months represents a medium-term observation period; therefore, late recurrence beyond 36 months cannot be ruled out, particularly in the absence of routine high-sensitivity postoperative imaging surveillance.

**Figure 7:**
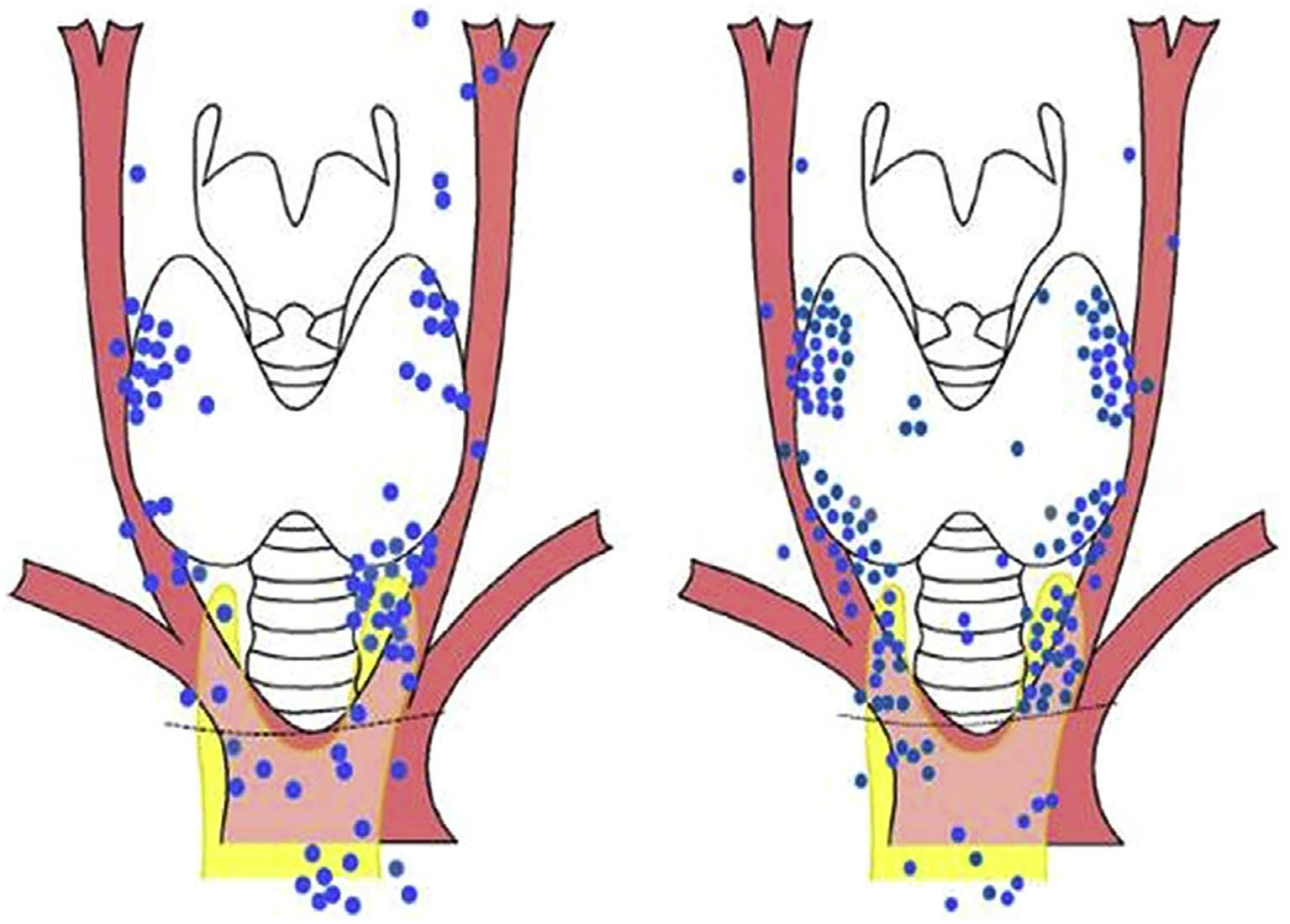
The results of the frequency distribution of ectopic glands by location (21).

### 3.7 Exploratory clinical outcomes

During the unified follow-up period, no patient in either group experienced pathological fracture, indicating that both surgical strategies effectively improved bone metabolism in SHPT patients. Cardiovascular events occurred in 3 patients (3.06%) in the Observation Group and 2 patients (2.5%) in the Control Group; all patients were successfully treated and discharged with no long-term neurological or cardiac dysfunction.

## 4 Discussion

SHPT is a key complication of end-stage CKD that severely impairs patient quality of life and increases mortality risk (20). Although standardized medical therapy can control mild to moderate SHPT, drug-refractory uncontrolled SHPT (iPTH > 800 pg/mL) requires surgical intervention due to the limited efficacy of pharmacotherapy and the high risk of irreversible complications such as renal osteodystrophy and cardiovascular calcification (3, 4). TPTX+AT is the mainstream surgical strategy for SHPT, but its postoperative recurrence rate remains a major clinical challenge (10–12). This challenge is mainly caused by residual ectopic parathyroid tissue that cannot be identified and resected during conventional surgery (15, 16).

To address this issue, we developed the CPTX+AT technique, an optimized surgical strategy that emphasizes systematic en bloc resection of the bilateral central neck compartment fibrofatty tissue (including the superior half of the thymus) on the basis of complete *in situ* parathyroid gland resection. In a separate study of recurrent SHPT patients by Okada et al. (21) **Figure 7**, ectopic glands were most frequently located in the mediastinum (22/93, 23.7%) and thymic tongue (31/146, 21.2%) respectively, while the central neck compartment contained a smaller proportion. Because the mediastinum lies outside the standard cervical surgical field, CPTX+AT specifically targets the accessible central neck and superior thymus, which together harbor the largest fraction of resectable ectopic tissue. The moderate sensitivity of conventional imaging for ectopic parathyroid glands (16/22, 72.7% in this series) underscores the importance of systematic surgical exploration, as nearly one-third of ectopic glands would have been missed by imaging alone. A key technical refinement of CPTX+AT is the selective resection of the superior half of the thymus instead of complete thymectomy—this aligns with contemporary evidence that routine complete thymectomy does not reduce SHPT recurrence but increases the risk of permanent hypoparathyroidism (22), balancing surgical radicality and safety.

In this study, all 98 patients who underwent CPTX+AT achieved surgical success. Although the Observation Group had a significantly longer operative time than the Control Group, this was considered a reasonable trade-off for the additional central neck dissection and ectopic gland resection, and it did not translate into a higher perioperative complication rate. ioPTH monitoring (≥90% decline from baseline) was used in all operations to confirm the completeness of resection. The overall complication rate in the CPTX+AT group was 12.24%, which is comparable to complication rates reported for parathyroidectomy in the setting of secondary hyperparathyroidism in large-scale studies (8), supporting the safety of this approach.

No severe complications—permanent hypoparathyroidism, permanent recurrent laryngeal nerve (RLN) injury, or hematoma—were observed in the Observation Group. This favorable safety profile was attributed to three key factors (1): meticulous surgical technique with intraoperative nerve monitoring (IONM) to protect the RLN (2); selective resection of the superior half of the thymus rather than complete thymectomy; and (3) a standardized postoperative protocol of high-dose 1,25-dihydroxyvitamin D and calcium supplementation, which effectively reduced the incidence of severe hypocalcemia and hypoparathyroidism. This aggressive supplementation strategy is consistent with current evidence emphasizing the importance of preventing postoperative hypocalcemia and hungry bone syndrome in SHPT patients (23, 24). It should be noted, however, that routine postoperative laryngoscopy was not performed in this study. As a result, transient hoarseness or subclinical RLN dysfunction may have been under-reported, which is a well-recognized limitation of retrospective surgical series. Future prospective studies should incorporate systematic pre- and postoperative laryngoscopic evaluation to accurately determine RLN injury rates following extended central neck dissection.

The most striking finding of this study was that no recurrence was detected during the median 25-month follow-up of the Observation Group, which was significantly superior to the 5.0% recurrence rate observed in the Control Group (p = 0.026). Kaplan-Meier analysis further confirmed the superior recurrence-free survival of the CPTX+AT group (*p* = 0.026). Notably, this result compares favorably to the 7–8% pooled recurrence rate of TPTX+AT reported in the literature (8). The fundamental reason for this improvement is that CPTX+AT, through systematic central neck compartment dissection, may more effectively eliminate residual ectopic parathyroid tissue—a major source of recurrence. In the Observation Group, 22 ectopic parathyroid glands were identified within the resected central neck compartment tissue, 45.45% of which showed hyperplasia. These ectopic glands would have been left *in situ* during conventional TPTX+AT and could have eventually led to SHPT recurrence under continued uremic stimulation (15, 16, 25).

From a biochemical perspective, CPTX+AT achieved rapid and durable normalization of calcium-phosphorus metabolism and iPTH levels, with all parameters stabilizing within the normal range by 6 months postoperatively. Clinically, complete resolution of symptoms was observed in all patients of the Observation Group by 3 months, leading to a marked improvement in quality of life. This is consistent with previous evidence that parathyroidectomy can rapidly alleviate SHPT-related symptoms (26). In addition, CPTX+AT did not compromise thyroid function: 97.96% of patients maintained normal FT3, FT4, and TSH levels throughout follow-up, which is attributable to the preservation of thyroid blood supply and parenchymal integrity during surgery (27).

Exploratory analysis revealed no significant differences between the two groups in mortality, fracture, or cardiovascular events. All deaths were unrelated to SHPT or surgery, indicating that CPTX+AT does not increase the risk of long-term adverse events. Owing to the scarcity of donor kidneys, no patient in either group underwent kidney transplantation during follow-up. These findings confirm that CPTX+AT is a safe option for patients who are not immediate transplant candidates and suggest that parathyroid function would remain normal should a transplant become possible later.

Several important methodological limitations should be acknowledged. First, the non-concurrent retrospective design: the two groups were recruited in different time periods, introducing potential temporal bias from improvements in surgical experience, perioperative care, and patient selection. Crucially, the median follow-up was substantially longer in the Control Group (47 months) than in the Observation Group (25 months) due to the non-concurrent design. This imbalance inflates the opportunity to observe recurrence in the Control Group and represents the greatest threat to the validity of the recurrence comparison. The reported 0% vs. 5% recurrence rates should therefore be interpreted with appropriate caution. Important variables that may influence recurrence risk—including dialysis duration, preoperative medical therapy, baseline iPTH severity, and surgeon learning-curve effects—were not adjusted for in multivariable analyses. The low event rate (n = 4) precluded meaningful propensity score matching or Cox regression. Third, the absence of high-sensitivity imaging such as ¹⁸F-fluorocholine PET/CT represents a major limitation. Although ⁹⁹^m^Tc-MIBI SPECT/CT and ultrasound are standard in resource-limited settings, their lower sensitivity for small or ectopic parathyroid glands, particularly in the mediastinum or retroesophageal space (28), means that the 22 ectopic glands identified intraoperatively in the CPTX+AT group may be an underestimate, and some residual ectopic tissue could have been present but undetected. Consequently, the reported 0% RLN injury rate should be interpreted with caution because routine postoperative laryngoscopy was not performed; transient or subclinical nerve dysfunction may have been underreported. Finally, although the median follow-up of 25 months is acceptable for medium-term outcomes, SHPT recurrences can occur beyond 3–5 years, especially from slow-growing autografted or residual ectopic tissue. Thus, our finding of “no recurrence” reflects the current observation period and does not guarantee lifelong disease eradication. Future prospective studies incorporating advanced imaging, systematic laryngoscopic evaluation, and extended follow-up (≥5 years) are essential to validate the suggested superiority of CPTX+AT.

## 5 Conclusions

CPTX+AT is a safe, feasible, and effective medium-term optimized surgical strategy for uncontrolled secondary hyperparathyroidism (iPTH > 800 pg/mL) in non-kidney transplantation candidates. Compared with conventional TPTX+AT, it achieves durable biochemical remission, complete resolution of clinical symptoms, and preserved thyroid function, with a comparable safety profile. Its most impactful advantage is the absence of detectable recurrence during a median 25-month follow-up, directly addressing the primary cause of surgical failure—residual ectopic parathyroid tissue. High-dose 1,25-dihydroxyvitamin D supplementation effectively reduces the risk of severe hypocalcemia, and intraoperative PTH monitoring is essential to confirm surgical completeness. For experienced endocrine surgeons, CPTX+AT has a manageable learning curve and may reduce long-term healthcare costs by minimizing SHPT recurrence, aligning with value-based healthcare principles. However, longer follow-up (≥5 years) and multi-center prospective studies are needed to confirm the durability of these results, as recurrence can occur beyond the current observation period.

## Data Availability

The original contributions presented in the study are included in the article/supplementary material. Further inquiries can be directed to the corresponding author.
